# Development of Specific *Thinopyrum* Cytogenetic Markers for Wheat-Wheatgrass Hybrids Using Sequencing and qPCR Data

**DOI:** 10.3390/ijms21124495

**Published:** 2020-06-24

**Authors:** Ekaterina Nikitina, Victoria Kuznetsova, Pavel Kroupin, Gennady I. Karlov, Mikhail G. Divashuk

**Affiliations:** 1 Laboratory of Applied Genomics and Crop Breeding, All-Russia Research Institute of Agricultural Biotechnology, Timiryazevskaya str. 42, Moscow 127550, Russia; shhket@gmail.com (E.N.); vika-kuz367@yandex.ru (V.K.); pavelkroupin1985@gmail.com (P.K.); karlovg@gmail.com (G.I.K.); 2 Kurchatov Genomics Center—ARRIAB, All-Russia Research Institute of Agricultural Biotechnology, Timiryazevskaya str. 42, Moscow 127550, Russia

**Keywords:** DNA repeats, fluorescence in situ hybridization, real-time quantitative PCR, wheat-wheatgrass hybrids

## Abstract

The cytogenetic study of wide hybrids of wheat has both practical and fundamental values. Partial wheat-wheatgrass hybrids (WWGHs) are interesting as a breeding bridge to confer valuable genes to wheat genome, as well as a model object that contains related genomes of *Triticeae*. The development of cytogenetic markers is a process that requires long and laborious fluorescence in situ hybridization (FISH) testing of various probes before a suitable probe is found. In this study, we aimed to find an approach that allows to facilitate this process. Based on the data sequencing of *Thinopyrum ponticum,* we selected six tandem repeat (TR) clusters using RepeatExplorer2 pipeline and designed primers for each of them. We estimated the found TRs’ abundance in the genomes of *Triticum* *aestivum*, *Thinopyrum ponticum*, *Thinopyrum intermedium* and four different WWGH accessions using real-time qPCR, and localized them on the chromosomes of the studied WWGHs using fluorescence in situ hybridization. As a result, we obtained three tandem repeat cytogenetic markers that specifically labeled wheatgrass chromosomes in the presence of bread wheat chromosomes. Moreover, we designed and tested primers for these repeats, and demonstrated that they can be used as qPCR markers for quick and cheap monitoring of the presence of certain chromosomes of wheatgrass in breeding programs.

## 1. Introduction

Cultivated wheat is the second most important food crop, providing calories and nutrients for up to one third of the world population. Before the Green Revolution, wheat was cultivated mainly (with few exceptions) as landraces [1,2]. Local landraces (e.g., Banatka, Beloturka, Poltavka, Kubanka, Arnautka, and others for the former USSR) were more heterogeneous in genetic composition, which had a number of advantages: greater plasticity of the genomes that make up their high adaptivity to local conditions and tolerance to abiotic stress [3,4,5]. However, as a result of modern breeding, most landraces were replaced by high-yielding disease, and lodging resistant (but genetically homogeneous) cultivars. As a result, the genetic diversity of wheat shifted towards European germplasm, especially in Asian countries, and was accompanied by the loss of the local old varieties [2,5,6,7]. The introgression of agronomically valuable traits from the Triticeae wild species via wide hybridization has been proved to be one of the efficient tools to enrich wheat gene repertoire [6,8,9]. *Thinopyrum* (wheatgrass) species are widely used in wide hybridization of wheat as donors of tolerance to adverse conditions and disease resistance [10,11,12,13,14,15,16,17].

For introgression of the genes from wheatgrass, the partial wheat–wheatgrass amphidiploids, or hybrids (WWGH) are used. WWGHs carry a combination of wheat and wheatgrass genomes, which makes it possible to use them as breeding bridges to facilitate the transfer of genes of interest from wheatgrass to wheat [18,19,20,21,22,23]. Many partial WWGHs possess unique composition of wheatgrass chromosomes associated with agronomically valuable traits [24,25,26,27]. They have good baking qualities, are resistance to leaf rust, drought, and salinization. Thus, the study of WWGHs is important for both wheat breeding improvement and for the development of WWGH as a commercially cultivated crop, such as perennial wheat [28,29,30]. Moreover, studying WWGHs themselves provides an interesting source of knowledge about the genome stability and genetic interactions in amphidiploids [31,32,33,34,35,36].

One of the basic approaches in the study of WWGHs and introgression lines, along with molecular markers, remains cytogenetic studies with common and specific probes [21,37,38,39,40,41,42]. This approach enables the discrimination of related subgenomes in amphidiploids, visualization of chromosomal composition, and identification of rearrangements. However, cytogenetics is a rather cost- and labor-consuming technique, which limits its throughput. In addition, until recently, it was a challenge to find a high-copy satellite repeat that could be used as a probe for fluorescent in situ hybridization. Previously, we proposed, and successfully tested, a qPCR-based algorithm to quickly assess the suitability of probes for use in cytogenetics [43,44].

In this work, we performed sequencing of a *Thinopyrum ponticum* accession, which is possibly one of the parental forms for the WWGH accessions from the collection of the Tsitsin Main Botanical Garden of Russian Academy of Sciences (Moscow, Russia). A cluster analysis of the sequences was performed using the RepeatExplorer2 pipeline [45], and a set of tandem repeats was selected. Using qPCR, the specificity of these repeats for wheatgrass chromosomes in WWGH genomes was predicted and then verified using fluorescence in situ hybridization. As a result, we have proposed a number of cytogenetic and qPCR markers for chromosomes of wheatgrass origin in the genomes of WWGH.

## 2. Results

### 2.1. Bioinformatic Analysis of the Thinopyrum Ponticum Genome. The Search of the TRs and Primer Design

**Figure 1:**
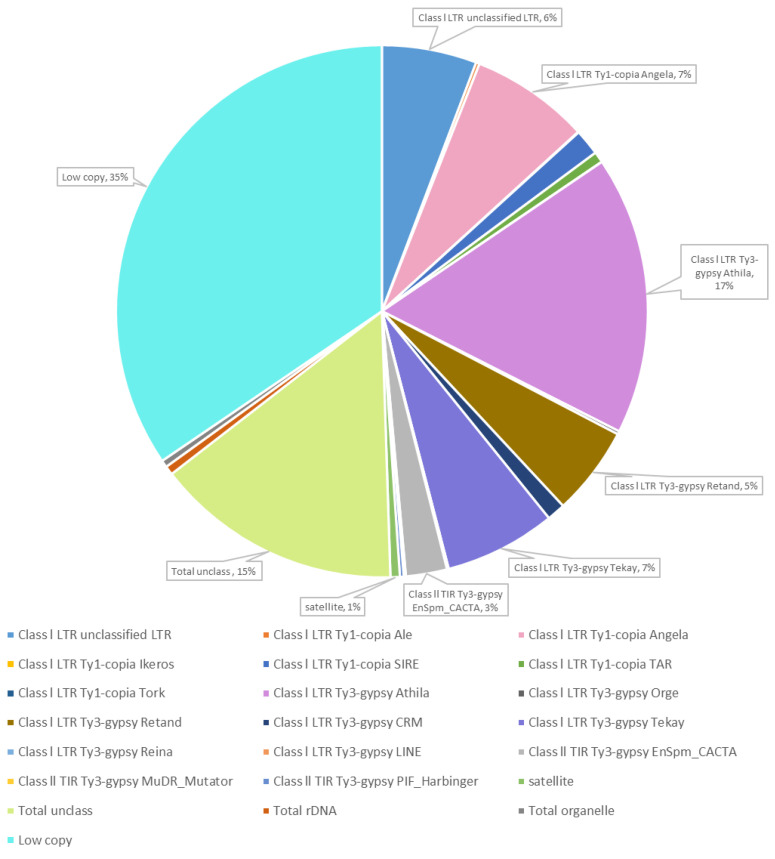
Proportion of repetitive DNA sequences of *Thinopyrum* *ponticum* identified after RepeatExplorer2 cluster analysis of whole-genomic Illumina sequencing data.

Whole-genome sequencing Illumina NextSeq platform of *Thinopyrum ponticum* DNA followed by computational processing using RepeatExplorer2 pipeline resulted in the data on the composition DNA repeats and its content in the *Th. ponticum* genome (Figure 1).

The repeated DNA accounted for 50% (the values are in % of all reads that were analyzed). The most of these repeats were mobile genetic elements (48.92%), and 0.57% were DNA satellites. Low-copy repeats amounted to 34.5% and unclassified repeats to 15%. Another 0.54% accounted for ribosomal DNA and 0.43% for DNA from organelles.

Among the mobile elements, the majority was classified as retroelements, their proportion was 46.06%. Of them, Athila (16.95%) and Angela (7.24%) were most common. The share of transposons was 2.86% with a strong predominance of EnSpm_CACTA (2.5%).

Cluster analysis using the RepeatExplorer2 pipeline resulted in 24 clusters, defined as satellites, of which we selected 6 clusters for further analysis (Table 1), based on overall assessment by the following criteria: the copy number of clusters, the absence of homology to wheat repeats (or homology to small amount of wheat repeats), homology to the repeats revealed in other wild Triticeae species.

### 2.2. qPCR Analysis of the TANDEM REPEATs

qPCR analysis of tandem repeats was performed using DNA from four studied WWGH accessions, bread wheat, *Th. ponticum* and *Th. intermedium*. *VRN1* was used as a reference gene to normalize the level of tandem repeats in the tested DNAs.

**Figure 2:**
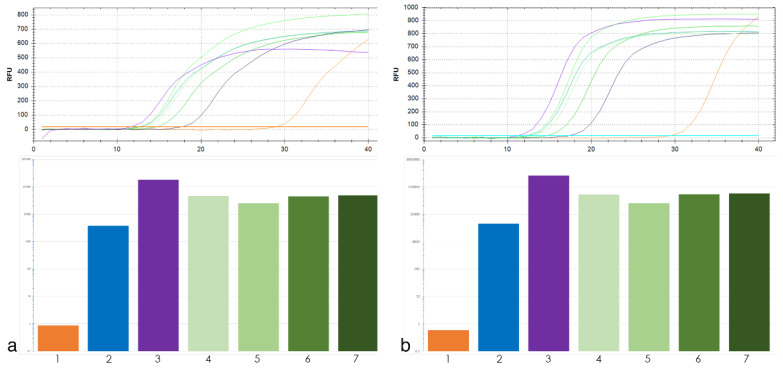
Top, amplification curves of 19-202 primers pair No. 1 (**a**) and No. 2 (**b**) tandem repeat. Colors of curves corresponds to target DNA: *T. aestivum* (orange), *Th. intermedium* (violet), *Th. ponticum* (blue), wheat–wheatgrass hybrid (WWGH) (shades of green). Bottom, the histogram of copy number 19-202 tandem repeat: 1—*T. aestivum*, 2—*Th. ponticum*, 3—*Th. intermedium*, 4—WWGH 166, 5—WWGH 548, 6—WWGH 4044, 7—WWGH ZP26.

For qPCR analysis of tandem repeat 19-202 two pairs of primers were used. The qPCR results were similar for both primer pairs (Figure 2). Thus, the exact primer location within the locus of interest was not critical for the copy number assessment of the tandem repeat using qPCR.

The results of qPCR analysis are as followed.

Tandem repeats 19-202 and 17-62 did not have high copy number in *T. aestivum*, but they had high copy number in *Th. ponticum* and *Th. intermedium* with approximately the same abundance in all four WWGHs (Figure 2). Repeat 19-202 analyzed by BLAST did not show homology to any *T. aestivum* repeats (Table 1). BLAST search did not reveal homology of repeat 17-62 to satellite repeats in wheat, but displayed homology to its transposable elements and different bacterial artificial chromosomes (BACs). However, the qPCR assay has clarified the results of bioinformatic analysis and demonstrates feasibility of 17-62 as a FISH probe.

Tandem repeat 17-251 had more than one hundred-fold higher copy number in wheatgrass than in *T. aestivum*. The WWGH accessions showed a copy number level of 17-251 close to wheatgrass. (Figure S1). Tandem repeat 17-202, which has partial homology to the CentT550 repeat, had about 10-fold higher copy number in *Th. intermedium* compared to *T. aestivum*, and its abundance in the WWGHs was at an intermediate level (Figure S1). Repeats 17-172 and 18-158 (Figure S1) had similar copy number in *T. aestivum*, *Th. intermedium*, *Th. ponticum* and the WWGHs. Bioinformatic analysis showed the homology of 18-158 between wheatgrass and bread wheat, while 17-172 displayed homology to the *Agropyron cristatum* tandem repeat and partial homology to wheat BACs (Table 1).

### 2.3. FISH/GISH Experiments

**Figure 3:**
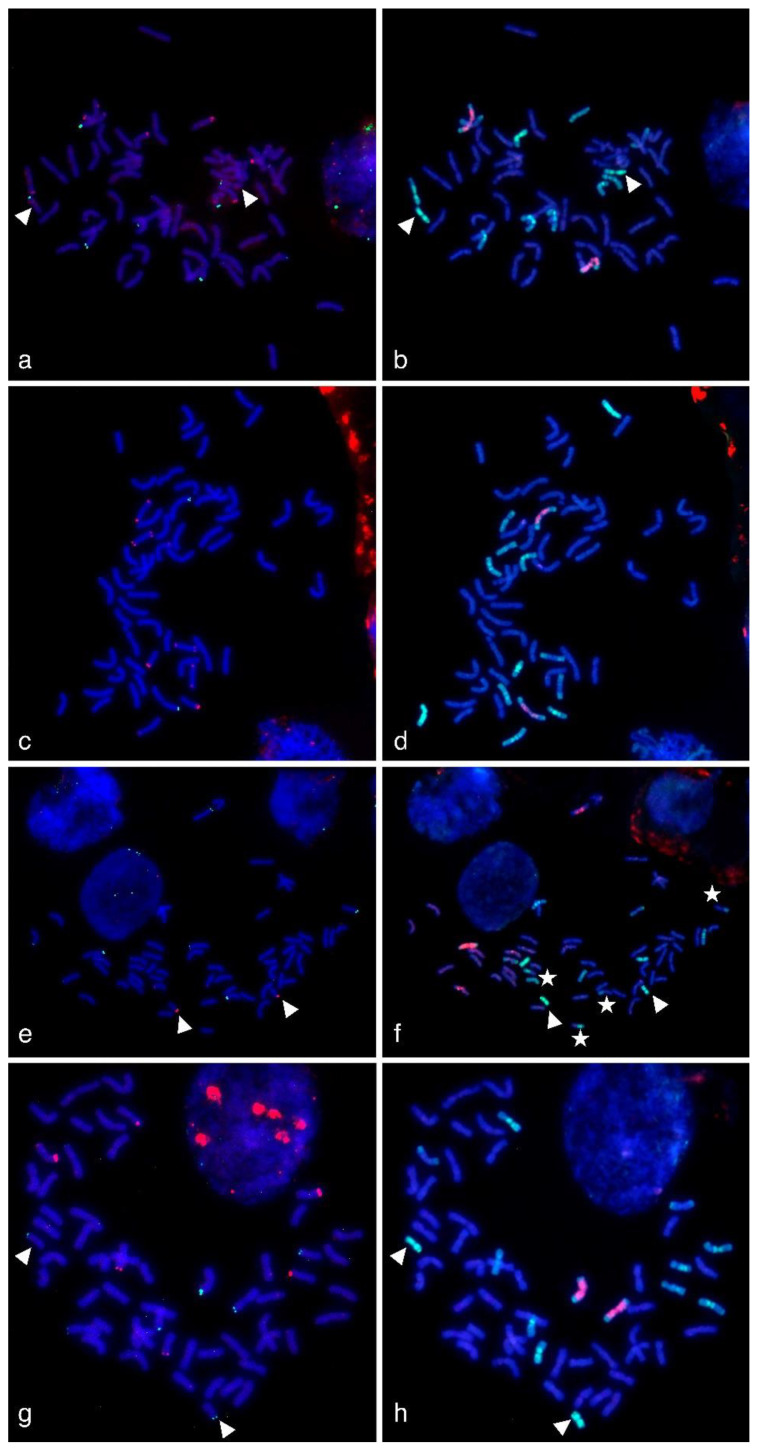
Multicolor-fluorescence in situ hybridization (mc-FISH) in the partial amphiploids WWGH 166 (**a**), WWGH 548 (**c**), WWGH 4044 (**e**), WWGH ZP26 (**g**), probed with 17-251 (green, digoxigenin) and 17-62 (red, biotin), and genomic in situ hybridization (GISH) on the same chromosome spread with total genomic DNA of *P. spicata* (green) and *D. villosum* (red) as probes (**b**,**d**,**f**,**h**). Chromosomes counterstained with DAPI (blue). Arrowheads indicate short St chromosomes; white stars indicate translocated chromosomes in WWGH 4044.

**Figure 4:**
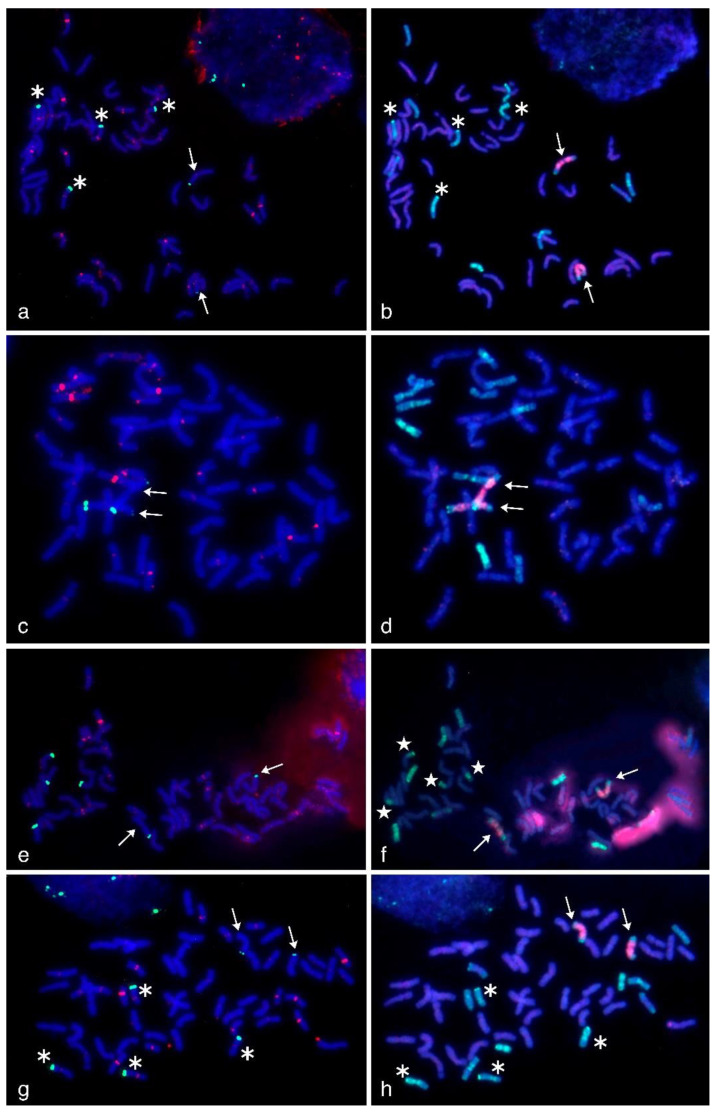
Multicolor-fluorescence in situ hybridization (mc-FISH) in the partial amphiploids WWGH 166 (**a**), WWGH 548 (**c**), WWGH 4044 (**e**), WWGH ZP26 (**g**) probed with 17-202 (green, digoxigenin) and probe 19-202 (red, biotin) and genomic in situ hybridization (GISH) on the same chromosome spread with total genomic DNA of *P. spicata* (green) and *D. villosum* (red) as probes (**b**,**d**,**f**,**h**). Chromosomes counterstained with DAPI (blue). Arrows indicate J^vs^ chromosomes; asterisks indicate chromosomes with simultaneous localization of 17-202 and 19-202 probes; white stars indicate translocated chromosomes in WWGH 4044.

The probes that were tested in qPCR analysis were evaluated as FISH probes. The probes were applied to the metaphase plates of WWGH that contained both wheat and wheatgrass chromosomes. Additionally, we performed sequential multi-color GISH using labeled genomic DNA of *Pseudoroegneria spicata* and *Dasypyrum villosum* as probes. It enabled the identification of wheatgrass chromosomes and discrimination of them from wheat chromosomes. We revealed, that chromosome composition of the studied accessions was 42W + 14WG for WWGH 166, WWGH 548, and WWGH ZP26, and 40W + 16WG, for WWGH 4044. In WWGH 4044, two pairs of chromosomes had intergenomic translocation (Figure 3 and Figure 4, shown with white stars). Wheatgrass chromosomes after GISH procedure differed from each other by the probe hybridization pattern.

The comparison of the same metaphase plates after FISH and sequential GISH procedure enabled us to localize DNA repeats 17-251, 17-62, 17-202, and 19-202 to wheatgrass chromosomes as follows.

In general, repeat 17-62 was localized to the terminal (subtelomeric) regions in two or three pairs of wheatgrass chromosomes on one or both arms (red signal, Figure 3). In WWGH 166, it was localized to three pairs of wheatgrass chromosomes. In WWGH 548, it was localized to three pairs of wheatgrass chromosomes, one pair had signal on one arm, two chromosomes had signals of different intensity on both arms. In WWGH 4044, 17-62 was localized to two pairs of wheatgrass chromosomes: the signal on one pair was much stronger than on another. In WWGH ZP26, it was localized to three pairs of wheatgrass chromosomes. Two pairs of chromosomes had signals on one arm, the other chromosome pair the strongest signal on one arm and the minor signal on another arm.

In general, repeat 17-251 was localized to the terminal (subtelomeric) regions in one, two or three pairs of wheatgrass chromosomes on one arm (green signal, Figure 3). In WWGH 166, it was localized to three pairs of wheatgrass chromosomes. In WWGH 548, it was localized to one pair of wheatgrass chromosomes. In WWGH 4044, 17-251 was localized to two pairs of wheatgrass chromosomes: the signal on one pair was much stronger than on another. In WWGH ZP26, it was localized to three pairs of wheatgrass chromosomes. In FISH metaphase plates with repeat 17-251, minor signals on some wheat chromosome pairs were also observed (Figure 3). It could be explained by its partial homology to Spelt52.2 localized to wheat chromosomes [46,47]. However, the FISH probes developed by PCR with primers for 17-251 and DNA of *Th. ponticum* produced strong major signals in wheatgrass chromosomes and just minor signals in wheat chromosomes.

In general, repeat 17-202 was localized to the pericentromeric regions in five or six pairs of wheatgrass chromosomes (red signal, Figure 4). In WWGH 166, it was localized to six pairs of wheatgrass chromosomes. Chromosome pair with a weak 17-202 signal additionally had subtelomeric signal of repeat 19-202. In WWGH 548, it was localized six pairs of wheatgrass chromosomes, in two pairs of them the signal was very strong. In WWGH 4044, 17-202 was localized to five pairs of wheatgrass chromosomes, of which one pair had the most intensive signal. In WWGH ZP26, it was localized to six pairs of wheatgrass chromosomes, the signal differed in its intensity between pairs of chromosomes, one pair was characterized by the strongest signal. In all WWGH metaphase plates, the pericentromeric signal was registered in some wheat chromosomes as well. 

In general, repeat 19-202 was localized to the terminal (subtelomeric) regions in one or three pairs of wheatgrass chromosomes on one or both arms (green signal, Figure 4). In WWGH 166, it was localized to three pairs of wheatgrass chromosomes, two pairs had the signal more intensive than the other one. In WWGH 548, it was localized to one pair of wheatgrass chromosomes on both arms; on one arm the signal was stronger than on another. In WWGH 4044, 19-202 was localized to three pairs of wheatgrass chromosomes. In WWGH ZP26, it was localized to three pairs of wheatgrass chromosomes, two pairs had signal stronger than the other pair.

In general, repeats 17-251, 17-62, and 19-202 demonstrated terminal (subtelomeric) localization on few wheatgrass chromosomes, while 17-202 was hybridized to pericentromeric regions of the majority of wheatgrass chromosomes. All studied repeats reproducibly differed in their signal intensity between wheatgrass chromosome pairs. 

Although, in general, repeats 17-251, 17-62, and 19-202 were specific for wheatgrass chromosomes, in certain metaphase plates distinct signals were observed on wheat chromosomes as it was revealed by sequential GISH procedure (Figure S2). It could be either due to partial homology of the found repeats to wheat DNA or to the local FISH conditions on the slide (e.g., stringency washing). We considered it necessary not only to demonstrate the clean results in our study but also to point out the possible signals in wheat chromosomes in order to note the potential problems of non-specific hybridization at FISH optimization.

WWGH had its own specific pattern of GISH/FISH signals in chromosomes that enabled wheatgrass chromosome identification. For example, a large chromosome with *D. villosum* DNA (V genome) probe in the proximal regions (red signal) and *P. spicata* DNA (St genome) probe in the terminal regions (green signal) can be classified as J^vs^ chromosome (shown with arrows in Figure 4). Repeat 19-202 (green signal) was localized to one arm of this chromosome in accessions WWGH 166, WWGH 4044, and WWGH ZP26, while in WWGH 548 it was hybridized to both arms (shown with arrows in Figure 4). Other example of the specificity of the developed probes is the shortest centromeric chromosome with the bright St genome signal along its length that can be classified as St chromosome (shown with arrowheads in Figure 3). In accessions WWGH 166 and WWGH ZP26 in the terminal region of this chromosome repeat 17-62 was localized (green signal), while in WWGH 4044 a clear signal of repeat 17-251 is observed (red signal). Moreover, in two accessions, WWGH 166 and WWGH ZP26, repeats 17-202 (in pericentromeric region, minor red signal) and 19-202 (in terminal region, major green signal) were localized simultaneously to two chromosome pairs, that was not observed in WWGH 4044 and WWGH 548 (shown with asterisks in Figure 4).

Repeat 17-172 in WWGH 166 was localized to 27 pairs of chromosomes, mainly in the subtelomeric region. The signal intensity strongly varied between different chromosomes. In WWGH 548, the repeat was localized on 22 pairs of chromosomes mainly in the subtelomeric region. In WWGH 4044 the repeat was localized on all pairs of chromosomes. In WWGH ZP26, the repeat was localized on 26 pairs of chromosomes (red signal, Figure S3). Thus, this repeat is localized both on wheat and wheatgrass chromosomes in the WWGH genome. The comparison of FISH and sequential GISH metaphase plates showed that repeat 17-172 produces more strong signals on wheat chromosomes rather than on wheatgrass chromosomes. This was predicted by qPCR, but bioinformatic analysis did not give such a prediction, since it did not show homology between wheatgrass and wheat repeats. Most likely, the primers used for qPCR for this tandem repeat are able to amplify several repeat sequences, or one of the most common repeats in cereals, such as homologues of pAs1, pSc119, etc.

Repeat 18-158 in WWGH 166 was localized on 27 pairs of chromosomes, mainly in the subtelomeric region. The signal intensity strongly varied between different chromosomes. In WWGH 548, the repeat was localized on 22 pairs of chromosomes mainly in the subtelomeric region. In WWGH 4044, the repeat was localized on all pairs of chromosomes. In WWGH ZP26, the repeat was localized at 26 pairs of chromosomes (green signal, Figure S3). Thus, this repeat is localized both on wheat and wheatgrass chromosomes in the WWGH genome. The sequential GISH on the same metaphase plate showed that repeat 18-158 produces more strong signals on wheat chromosomes than on wheatgrass chromosomes. At the same time, its chromosome localization is very similar to the localization of 17-172. Although the localization of the 18-158 repeat in both wheat and wheatgrass chromosomes was not predicted by standard bioinformatics analysis, qPCR analysis was able to predict this possibility.

## 3. Discussion

The development of cytogenetic markers is a tedious process that requires long and laborious FISH testing of various probes before a suitable probe is found. In many cytogenetic studies aimed at molecular-cytogenetic characterization of wheat–wheatgrass hybrids, the classical FISH probes such as 45S rDNA, pAs1, pSc119.2, and pTa71 are applied [32,37,38,39,40,41,42]. However, the development of new *Thinopyrum*-specific probes is necessary as it would enable the precise identification of chromosome rearrangements in amphidiploids and introgression lines and can be used in evolutionary studies of Triticeae [40,43,48,49].

The DNA repeats are one of the most prominent genome fractions to be converted into FISH probes. The development of whole-genome sequencing and in silico analysis greatly facilitated the search for repeated DNA sequences. Having performed shallow whole genome sequencing in *Th. ponticum*, we analyzed repeated DNA composition including mobile DNA. The obtained results contribute to the general understanding of the distribution of transposable elements in grasses genomes [50,51,52].

In this study, we aimed to find an approach that allows to facilitate and fasten the process of wheatgrass-specific cytogenetic markers; [52] developed *Thinopyrum*-specific FISH probes by performing SLAF-seq technology with subsequent selection of genome-specific repeated DNA by the bioinformatic analysis. Moreover, [40,53] added conventional PCR to this approach to estimate the specificity of the sequences in order to reveal the specific FISH probes. We introduced qPCR step as a new chain in this pipeline, which enables the direct estimation of repeat copy number and its specificity among genomes [43]. We assumed the repeats that vary strongly in copy numbers between wheat and wheatgrass chromosomes would be good cytogenetic markers for WWGH. Using shallow whole genome sequencing followed by bioinformatic sequence analysis and qPCR, we compared the relative copy numbers of selected tandem repeats in wheat, wheatgrass, and WWGH, and were able to determine tandem repeats that work well as cytogenetic markers.

As expected, a high copy number of the repeats corresponded to a high signal intensity in FISH procedure. An introduction of additional step, qPCR, further improved the development of cytogenetic markers using bioinformatics approach. qPCR is a high throughput and affordable technique compared to cytogenetics and sequencing with bioinformatics analysis. In this study qPCR step was informative enough to exclude repeats 17-202, 17-172 and 18-158 from consideration as targets for cytogenetic markers, and it was confirmed true by FISH. Potentially, qPCR can also help with preliminary differentiation of samples for analysis if contrasting lines are necessary to determine. This scenario can be illustrated by WWGH 548 accession in this work, which showed the lowest relative copy number among the other WWGH accessions for 19-202 repeat (Figure 4), and had less amount of FISH signals compared to other WWGHs: WWGH 548 had totally four signals (on both arms of a pair of wheatgrass chromosomes), while the remaining accessions had six signals (on one arm of three chromosome pairs (Figure 4).

The results of the in situ hybridization experiments showed that four studied WWGHs differ in the composition of wheatgrass chromosomes and, accordingly, are potential sources of different genes of agronomically valuable traits for introgression in wheat genome. Indeed, in our previous studies, as well as in the breeder estimations, the studied WWGHs accessions demonstrated the differences in bread-making quality, post-harvest regrowth, perenniality, seed storage proteins composition, resistance to leaf rust, salt tolerance, resistance to pre-harvest sprouting, and other traits [25,26,54,55,56]. Additionally, molecular studies demonstrated that the four studied WWGHs has different allelic state of the wheatgrass genes that are associated with the studied traits [26,55].

Amplification polymorphisms in multi-locus AFLP markers demonstrated the closeness of WWGH ZP26 and WWGH 166 [29]. Our results coincide with these observations. Indeed, WWGH ZP26 and WWGH 166 demonstrated very similar pattern of GISH/FISH signals in wheatgrass chromosomes. Nevertheless, it should be noted that WWGH ZP26 and WWGH 166 have different phenotype characteristics (postharvest regrowth, pre-harvest sprouting) and allelic state of certain genes, and, thus, cannot be considered as one and the same line. 

The analysis using genome-wide PCR markers (iPBS, RAPD, ISSR) revealed the genetic closeness of WWGH ZP26 and WWGH 4044 [56]. Here, we showed that the chromosome composition of these WWGHs is rather contrast. We can assume that WWGH ZP26 and WWGH 4044 were selected from the same cross but segregation in wheatgrass chromosomes with subsequent artificial selection resulted in different chromosome sets inherited from one and the same germplasm. WWGH 4044 has an unusual wheatgrass chromosome composition, 40 wheat and 16 wheatgrass chromosomes, of which two pairs carried intergenomic translocations. The agronomic performance of the revealed translocations can be assessed and further used in breeding process using our chromosome markers. Therefore, the probes 17-251, 17-62, and 19-202 revealed in this study can be considered as verified and robust markers for wheatgrass chromosomes.

## 4. Materials and Methods

### 4.1. Plant Material

The following accessions of wild grasses were used in the study: *Thinopyrum intermedium* PI 383573 (2n = 6x = 42, J^r^J^vs^St), *Thinopyrum ponticum* (=*Thinopyrum elongatum*) 1158A/19 (2n = 10x = 70, JJJJ^s^J^s^). The following accessions of wheat-wheatgrass hybrids (WWGH) were used: 4044, 548, ZP26, 166. Common wheat accessions cv. Nemchinovskaya 56 were used as controls. Accessions PI were ordered via Germplasm Resources Information Network (National Genetic Resources Program of USDA). *Th. ponticum* accession 1158A/19 (kindly provided by Dr. L.I. Glukhova) and WWGH accessions (kindly provided by Dr. V.I. Belov) were developed at the Department of Distant Hybridization, Tsitsin Main Botanical Garden of Russian Academy of Sciences, Moscow, Russia.

### 4.2. Sequencing and Bioinformatics Analysis of Thinopyrum Ponticum 1158A/19

DNA was isolated from an accession of *Thinopyrum ponticum* 1158A/19, using the CTAB method [57]. Whole-genome libraries were carried according Swift 2S Turbo protocol (Swift Bioscience, Ann Arbor, MI, USA); starting amount of DNA was 25 ng, the length of fragments ~350 bp; pair-end index from Swift 2S Turbo Unique Dual Indexing Kit. The run was performed with Illumina protocols on the Illumina NextSeq with NextSeq 500/550 Mid Output Kit v2.5 (300 cycles) with pair-end reads. The length of read was 151 bp, the length of index was 8 bp. Illumina sequencing was ordered in Genomed, Ltd. (Moscow, Russsia).

The result was 15,232,480 pair-end reads. Sequencing reads were analyzed by quality control tool FastQC (https://www.bioinformatics.babraham.ac.uk/projects/fastqc/ (accessed on 15 June 2020)), followed by quality filtering based on the sequence quality score, adaptors trimming, filtering reads shorter than 100 bp or unpaired sequences using the Trimmomatic tool [58]. Quality-filtered reads were randomly sampled to 10,000,000 paired-end reads of which 4 752 527 were analyzed using RepeatExplorer2 pipeline [45,59].

Genome coverage was calculated as follow: cov = (r * l)/g, where r corresponds to number of reads used in our analysis, l to read length and g to haploid genome size of *Thinopyrum ponticum* and amounted to 10.4%.

From the results of cluster analysis, we selected clusters according to the following criteria: (1) the automatic annotation RepeatExplorer2 identified them as satellites with a high degree of probability, (2) clusters have medium or high copy numbers (see Table 1). Then we performed additional alignment of the potential repeats using our own databases and public databases of known repeats using BLAST. The main criterion for this stage of new repeats selection was weak or absent homology to known wheat repeats.

### 4.3. Real-Time qPCR

qPCR was performed using as templates DNA of *T. aestivum*, *Th. ponticum*, *Th. Intermedium,* and four accessions of partial wheat-wheatgrass hybrids, WWGH 548, WWGH ZP26, WWGH 4044, and WWGH 166.

Primers for selected clusters were designed using Primer3 program (primer length 20 bp, Tm = 60 °C, see Table 2 for primer sequences). For cluster 19-202, two pairs of primers were selected for different repeat regions in order to evaluate their performance (Table 2). The qPCR was carried out using CFX Real-Time PCR Detection System (Bio-Rad) and Real-Time PCR Mix reaction mixture with Eva Green (Syntol Ltd., Moscow, Russia) according to the manufacturer’s protocol. Primers were synthesized at Syntol Ltd. (Moscow, Russia). The reference gene used was VRN1. The primer concentration was 10 ng/μL, and the DNA concentration was 0.4 ng/μL. The amplification program was as follows: pre-incubation for 10 min. at 95 °C, then 40 cycles: denaturation for 10 s at 95 °C; primer annealing for 30 s at 60 °C.

### 4.4. Fluorescence In Situ Hybridization (FISH) and Genome In Situ Hybridization (GISH)

The physical localization of the 6 tandem repeats (TRs) was analyzed by the FISH procedure. A chromosome spread preparation was made from root tip as described in [28]. Good slides with well-spread chromosomes were stored at 4 °C until use. FISH was carried out following the procedure in [60,61]. After hybridization, the chromosomes were counterstained with 1 mg/mL DAPI. The detection was performed using streptavidin-conjugated Cy3 or FITC (Roche, Basel, Switzerland); signals in all variants were visualized using an AxioZeiss Imager V1 fluorescence microscope.

After post-hybridization washing of WWGH chromosome mitotic preparations as described in Komuro et al. [62] multicolor genomic in situ hybridization (mcGISH) was conducted on the same slides as described in Kishii et al. [63] with modifications described in Salina et al. [15]. The probes were the *Pseudoroegneria spicata* (St genome) and *Dasypyrum villosum* (V genome) genomic DNA (50 ng/preparation) labeled with digoxigenin-11-dUTP and with biotin-16-dUTP, respectively, by nick translation, according to the manufacturer’s instructions (Roche, Germany). The chromosomes were counterstained with 1 mg/mL DAPI and mounted in Vectashild (Vector Laboratories, Peterborough, UK).

The results were recorded with an AxioCam Mrm Zeiss camera and contrasted using AxioVision software.

## 5. Conclusions

In this work, we obtained three tandem repeat cytogenetic markers (19-202, 17-62, 17-251) that specifically labels wheatgrass chromosomes in the presence of bread wheat chromosomes. Moreover, we designed and tested primers for these repeats, and demonstrated that they can be used as qPCR markers for quick and cheap monitoring of the presence of certain chromosomes of wheatgrass in breeding programs, for hybrid crosses, introgression, obtaining substitution and introgression lines, and other purposes.

## Figures and Tables

**Table 1:** Characterization of selected satellite clusters, based on amount of copy, identification by RepeatExplorer2 as satellite with high probability and weak homology to known wheat repeats.

Cluster Name	Proportion, %	Length, bp	Consensus Cluster Sequence	Homology to the Known Repeats
Putative satellites (high confidence)				
19-202	0.034	380	GATTTTACATACGTGCACACACAGGATCACATGCGCGGAAAATATCGAGCCCAAAAAGGGCCGTCTGGGCCCTCAAAAATGGCCTGCAGGATTGGAAAAAATGAAAGTTATCGCAATTACAGCTCAAATTTCGATGAGCCGGCACATCCCTTTGGTGTTCAGGTCCTGGGCGCCCCACCCATGTATGGATACAATAGGGTCTTAGGCCAACTCTCGCAAAGAAACGGTGATCAAAGAAAATACAAAAATCAACCTAGAGTCTGAATTGATCGAGACTCTCAAAGCAAGTGAGAACAAGCTCCGATTTCATCGTTTTCATAGGCGTTGGAAAGCTATTGGGGAATTGAGCTGATAGGACTTCTAGTTTTTGTGATTTGGAG	MG323511.1 *Agropyron cristatum* clone ACRI_TR_CL20 satellite sequence sequence
				KP231286.1 *Agropyron cristatum* isolate Z559 TRT1 repeat sequence sequence
17-251	0.013	376	GATGAAAAACAAAAGTTTTGGCAGTTAAAGTTTGAGATTCGATGGTGCCGTAGATCCGTTTGGTCATCAGGTGTTACGAGCCACACCCATGGATAGCTAGAATAGTGTGCTAGGCTCAGACTAGAGAAGAAACGGGGAACAAACTGATGAAAGAATTATCATACCGAGGGAATTCACTCAGAACTAACAAGAATGATAACGTGCTCCGACTGGAGCGGTTTAGGAACTGCTGGAAGGCTCTAGGGGAATAAAGATGGAATGACTTCTAATTTTTAGGTCCGTATGATTTGAAATACGTGCACACAAACCCCGAGAAAGCGTTCCTGGAAATCTCGGCCCAAAAAAGGACGTTTAGTAATTCTAAAAGGGACTACAG	AY117401.1 *Aegilops speltoides* subtelomeric repeat Spelt52.2 sequence
Putative satellites (low confidence)				
18-158	0.097	333	GCCACACCCCGGAGACGCGTGCCGCCTCTTCGCACATGCCACCACACTTCCAGACATGTATGAGGGCCCGGTGCGACGCTCCGGTGGCATTGCTACCCCCCAGGGCCCCCGTCCCGCCTAACCCTGGAACGGTTGACCACGAGATCTAGCCCTTTGACTTTCGCCGGACGGGCTTTGACCAGTGGACCTTTCCACCTGGTTGTGATAGGTCAGCCCATAGGAACACCTCGGAGCAACGTCCGGGCCAAACCCACAGCAAGATTCCCTCCGTGTCGACCCGACGCGTCCGTTTCCCCCCTCCAGGTGCCGGCGGCGGCGCCGTCCGTGAGGGGG	KC290905.1 *Triticum aestivum* clone pTa-465 FISH-positive repetitive sequence
17-172	0.08	662	ATTGGAAAACCTTCGCATTGTGTCATTATATGTGACCAAGTTACCAGGAAAAATAATAAACTTGTAATACGGCAATTATTTTAAAAAAGTGTTCTCAGAAATGAGCTATCATGTGTGAAGATTCATGGCTTTCAAGCCAAATGATCAATCTTATGGCCACATTCATGGCATAGTTTGTTCAAATGATCTCATATTGTGCACAAGGGTGCATCTTGGAATGGCAAACAATGTTGCCTAAGGAAGTTTTCATTTTCTTTGGACGAAAAATTCATTTTCCATTTTTCGAGTGCCCAAAATGAGTTTTTTTGTGAAGGACCTACCATATATTTGTTGCAAAATTGGACCAAATCAATTTTCTAAAATATTAGGCCATATTTAATGCACAATTGACCAAATGGTTGGGTGTCAAAAGTTTTGATCCACCTCTCGTGAAAAAGACAAATTTCCGCCGATTCAGTAGGAAGCGGGTCAAATTTGAACTGCAGCTGCCTCATAGTTTGCTCTTTATTTTTTCCAAAAATCATTTCTAGGTACATAAGTATCTATTTAATCAGAGAAACACCAAAAGTTTTCCAAGATTCAACCACTAGCTAGGAACGGTCATGCCCGCCGTTTTGACCGCATTTTGAAACGGGCATAAAAAATTCAAAAAAAATCAAAAA	MG323513.1 *Agropyron cristatum* clone ACRI_TR_CL80 satellite sequence
17-202	0.032	553	TTTGTAATGGAAGGATGGTGCATTGTTCTATATGTTATTGTCCATATATCAGTCCGTAGGTGAGCTCACGGAAGGGTGGTAGAGGGTGGCAGAGTATACTTCAAACATAAAATCATCCGAAACTCAATTTTACAAGCCGGATCTTGCCTCCGAAATGTTGTCGAAGCCGGCGAGTGGGTTACGGACGCATACAACTTTTCGTTGTGATCGTTTTGGCGGGTCATGGAGCTCCAATGGAGTTTTTATGGCCAAATTGTGGCCGTTTTATGGATACAACATCGCGGGACAGACCGTGAATACAACTTTCAGGGTAAGTTGATCGCACCGACGAGCCATCTTGCACCATTCGGAATGACCTATAATTTTTCGTGTGCATAAACAGAATGAGGATGAGCTGTTATGTACTTTATGATCCAAGAATAATGCATCCGCTGGTGAAAACGTGAGGGTGGTAGCGGGATGGGTGATAGACCATGTAAAGCATGAATTCTTGGGTTTCGATGCAACGAAGAGCCTCCTCCTTCTGTCGTGACTGAACCTGTAGTCTTTCTAG	MN161206.1 *Triticum aestivum* clone CentT550 satellite sequence
					KT724936.1 *Secale cereale* clone BAC 19H13l pSc250 retrotransposon TREP231, complete sequence; and satellite pSc250 sequence
Putative LTR elements				
17-62	0.48	534	TCTCAAAATTTCGTTTCCCGCCCAAAGTTTCGTCTCCCGCTCGAAATATCGTCTCGCGCCCGTTCATCATATTTTTATCCCTCCCTCCCATTTTTGTATTTATTATTTATTTATTTTCCGGGGAGACGCGGTGGCAATGGTGGACAACAACACACCCTACTTTGGCATGACGGCGAAATTTTCCCGCCCAAAATGACGAAAAAATGACAACGGCCGCGAGTGTCTCAAAGCGCTCCCGGAGGTGTAAAAACGGGTATAGGAACGTATCACGGAGTTTGGTGGCCCCCAATCCCGGGATGGTGTCGAAAATTAGGGGCAATACGGGCATTACTTTTGTACCGGGCAACGTAGGATGGCCTCGGGGATGACAACGCAACCGGGGCAAAGGGGCTCCGACCCGGGCGGCCACGGGCCCGTCGGAGAGGCCTCGTGGAGACGGCGACCCGGGAAGACTACCTTCCGCGTCCCGGGCGCGTCCGCACTACGTGCCCTCTCAAGCAAAACCGCAAACGACCGCATCTCTCTCTCTCTCTC	MG323514.1 *Agropyron cristatum* clone ACRI_TR_CL85 satellite sequence

**Table 2:** Primer sequences for the tandem repeats.

Tandem repeat	Primer sequence
17-62	F: 5′-TTGCCCCTATTTTTCGACAC-3′
	R: 5′-GTGGCAATGGTGAACAACAA-3′
17-251	F: 5′-CAGTTCCTAAACCGCTCCAG-3′
	R: 5′-AGATCCGTTTGGTCATCAGG-3′
17-202	F: 5′-TCTATCACCCATCCCGCTAC-3′
	R: 5′-AATTGTGGCCGTTTTATGGA-3′
19-202_1	F: 5′-CAGCTCAAATTTCGATGAGC-3′
	R: 5′-TTCCAACGCCTATGAAAACG-3′
19-202_2	F: 5′-CATCCCTTTGGTGTTCAGGT-3′
	R: 5′-CCAACGCCTATGAAAACGAT-3′
17-172	F:5′-TGCAAAATTGGACCAAATCA-3′
	R:5′-GAGGCAGCTGCAGTTCAAAT-3′
18-158	F:5′-GGAAAGGTCCACTGGTCAAA-3′
	R:5′-ACATGCCACAACACTTCCAC-3′

## Supplementary Materials

The following are available online at https://www.mdpi.com/1422-0067/21/12/4495/s1, Figure S1: Top, amplification curves of tandem repeat. Colors of curves corresponds to target DNA: *T. aestivum* (orange), *Th. intermedium* (violet), *Th. ponticum* (blue), WWH (shades of green). Bottom, the histogram of copy number tandem repeat: 1—*T. aestivum*, 2—*Th. ponticum*, 3—*Th. intermedium*, 4—WWH 166, 5—WWH 548, 6—WWH 4044, 7—WWH ZP26. Amplification curves and the histogram of copy number of tandem repeats 17-62 (a), 17-251 (b), 17-202 (c), 18-158 (d), 17-172 (e). Figure S2: Multicolor-fluorescence in situ hybridization (mc-FISH) in the partial amphiploids WWGH 548 (a) probed with 17-251 (green, digoxigenin) and 17-62 (red, biotin), WWGH ZP26 (c) probed with 17-202 (green, digoxigenin) and probe 19-202 (red, biotin) and genomic in situ hybridization (GISH) on the same chromosome spreads with total genomic DNA of *P. spicata* (green) and *D. villosum* (red) as probes (b,d). Chromosomes counterstained with DAPI (blue). The presence of non-specific and not reproducible hybridization sites in wheat chromosomes is demonstrated. Figure S3: Fluorescence in situ hybridization (mc-FISH) in the partial amphiploids WWGH 166 (a), WWGH 548 (d), WWGH 4044 (g), WWGH ZP26 (j) probed with 17-172 (green, digoxigenin), WWGH 166 (b), WWGH 548 (e), WWGH 4044 (h), WWGH ZP26 (k) probed with 18-158 (red, biotin) and genomic in situ hybridization (GISH) on the same chromosome spread with total genomic DNA of *P. spicata* (green) and *D. villosum* (red) as probes (c,f,i,l). Chromosomes counterstained with DAPI (blue).

## Abbreviations

MDPI	Multidisciplinary Digital Publishing Institute
DOAJ	Directory of open access journals
WWGH	wheat–wheatgrass hybrid
qPCR	quantitative polymerase chain reaction
BAC	bacterial artificial chromosome
FISH	fluorescence in situ hybridization
GISH	genomic in situ hybridization
mcGISH	multicolor genomic in situ hybridization
