# Supplementary figures and images for: Development of Specific Thinopyrum Cytogenetic Markers for Wheat-Wheatgrass Hybrids Using Sequencing and qPCR Data
Source: Int J Mol Sci. 2020 Jun 24;21(12):4495. doi: 10.3390/ijms21124495 (PMC7349979; doi:10.3390/ijms21124495)

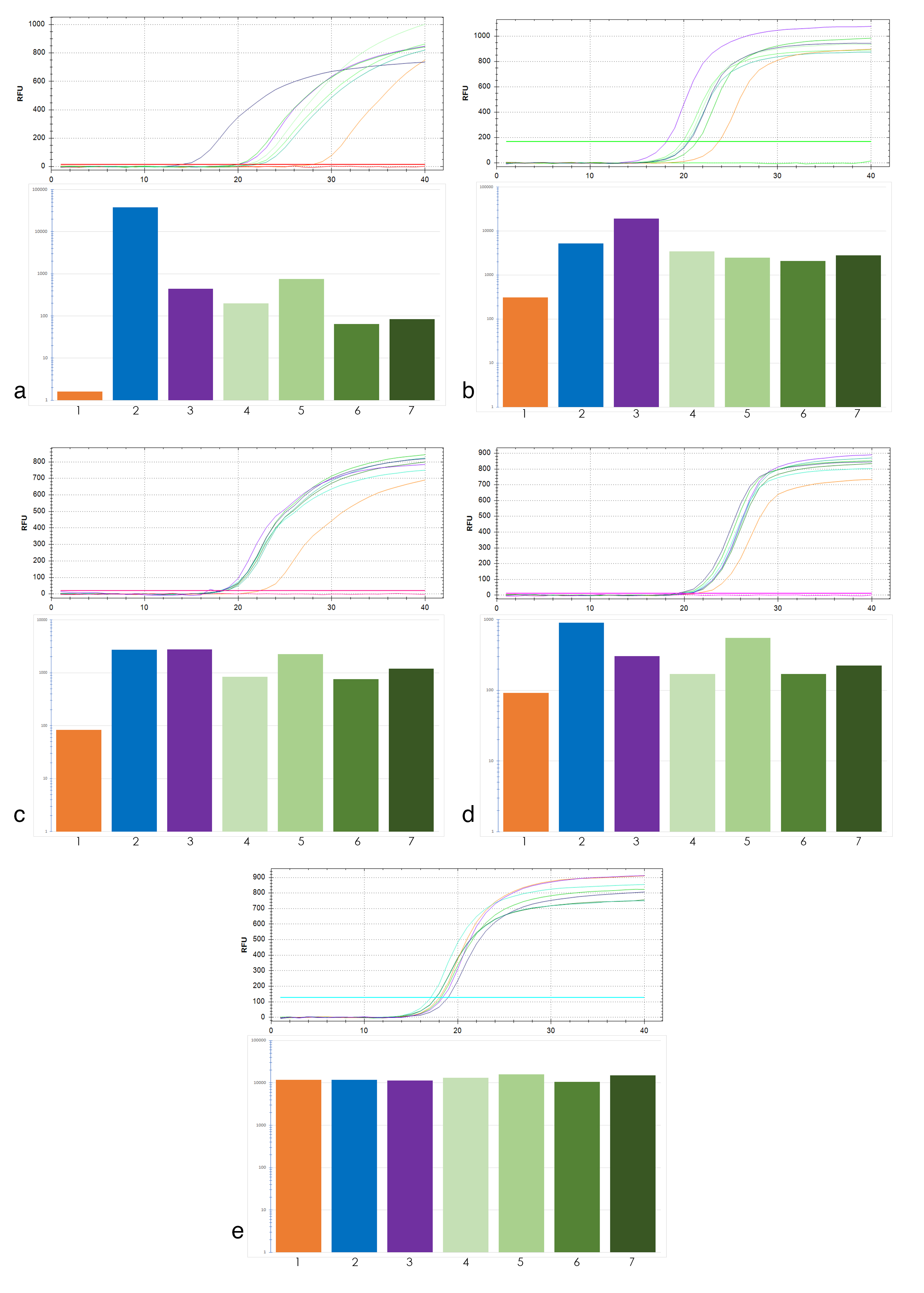

Supplement: Supplementary file 1 [file ijms-21-04495-s001.zip › Figure S1.jpg]

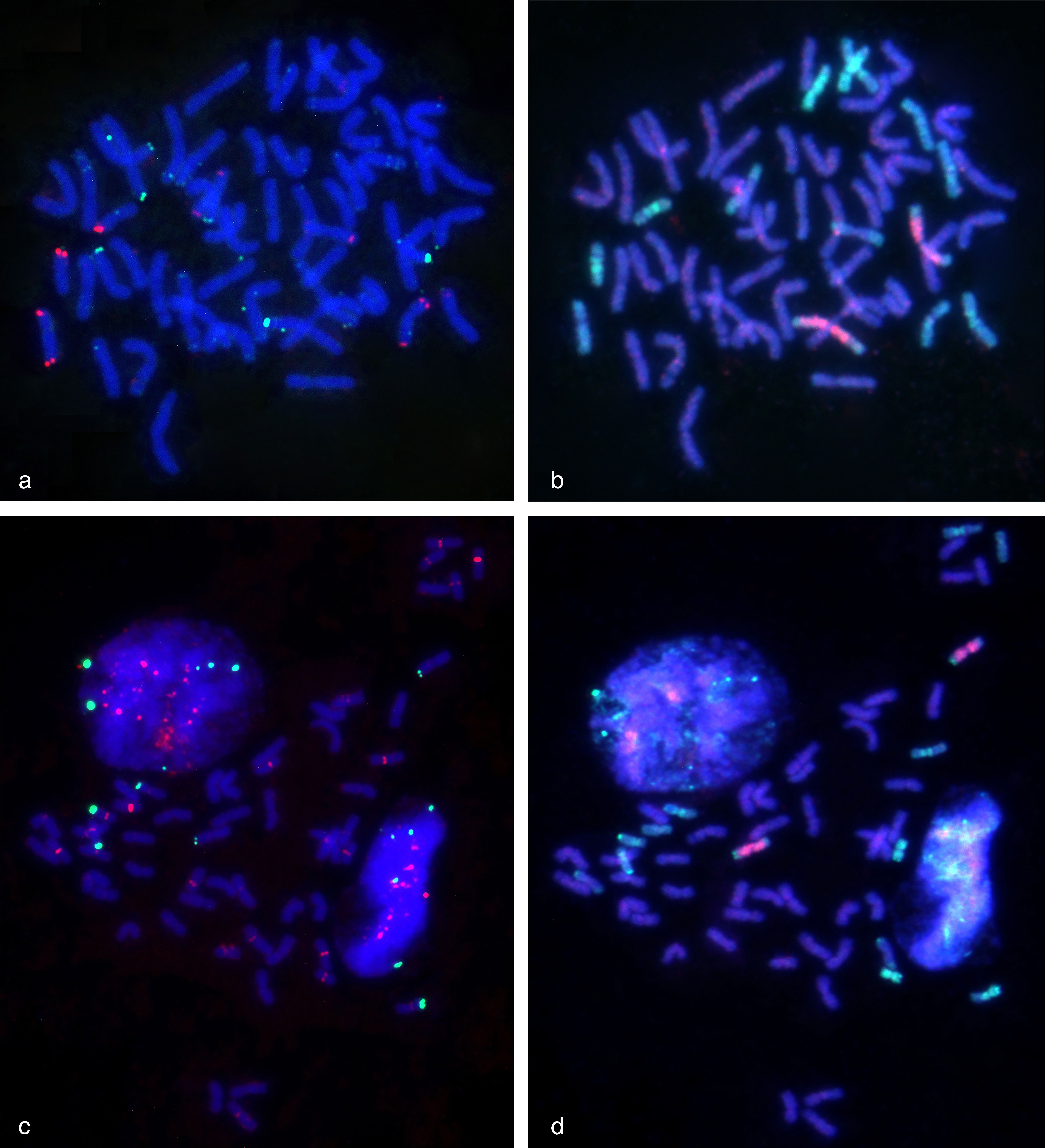

Supplement: Supplementary file 1 [file ijms-21-04495-s001.zip › Figure S2.jpg]
